# Conventional and Microcellular Injection Molding of a Highly Filled Polycarbonate Composite with Glass Fibers and Carbon Black

**DOI:** 10.3390/polym14061193

**Published:** 2022-03-16

**Authors:** Galip Yilmaz, Apichart Devahastin, Lih-Sheng Turng

**Affiliations:** 1 Technical Scientific Vocational School, Bayburt University, Bayburt 69000, Turkey; galipyilmaz@bayburt.edu.tr; 2 Wisconsin Institutes for Discovery, University of Wisconsin-Madison, Madison, WI 53715, USA; 3 Seagate Technology Company, Thepharak 10270, Thailand; apichart.devahastin@seagate.com; 4 Department of Mechanical Engineering, University of Wisconsin-Madison, Madison, WI 53706, USA

**Keywords:** microcellular injection molding, polycarbonate composite, flow marks, tiger stripes

## Abstract

Conventional solid injection molding (CIM) and microcellular injection molding (MIM) of a highly filled polycarbonate (PC) composite with glass fibers and carbon black were performed for molding ASTM tensile test bars and a box-shape part with variable wall thickness. A scanning electron microscope (SEM) was used to examine the microstructure at the fractured surface of the tensile test bar samples. The fine and uniform cellular structure suggests that the PC composite is a suitable material for foaming applications. Standard tensile tests showed that, while the ultimate strength and elongation at break were lower for the foamed test bars at 4.0–11.4% weight reduction, their specific Young’s modulus was comparable to that of their solid counterparts. A melt flow and transition model was proposed to explain the unique, irregular “tiger-stripes” exhibited on the surface of solid test bars. Increasing the supercritical fluid (SCF) dosage and weight reduction of foamed samples resulted in swirl marks on the part surface, making the tiger-stripes less noticeable. Finally, it was found that an injection pressure reduction of 25.8% could be achieved with MIM for molding a complex box-shaped part in a consistent and reliable fashion.

## 1. Introduction

Engineering polymer composites have been actively researched and developed for many applications. Composites allow tailoring material properties of interest, which is needed due to various product requirements in the polymer industry. For example, electrically conductive or static dissipative polymer composites are needed for handling and packaging electronic components [1,2,3,4]. Otherwise, those electronic components could be easily damaged by sparks resulting from electrostatic discharge (ESD) [5,6]. Nearly all neat polymers are not inherently ESD safe and must be modified by electrically conductive fillers [7].

Carbon-based fillers such as graphite, carbon fibers, carbon nanotubes, and especially carbon black are the most common conductive fillers [8,9]. Typically, more than 20 wt% of conductive filler is required for generating good conductivity [10]. However, highly filled composites with carbon black suffered from decreased deformability and toughness [10]. Although adding reinforcing fibers into the polymers enhance their mechanical properties [9,10,11], polymers heavily loaded with fillers and fibers present a processing challenge especially in injection molding. For example, high contents of solid fillers drastically increase the melt viscosity and result in high injection pressure and thermal degradation [12]. In some cases, fillers and fibers are not the only source of processing difficulties. Polycarbonate (PC), which can be used as the matrix polymer, is itself a very viscous polymer even at a high processing temperature. Nonetheless, PC yields quality products with great toughness and dimensional accuracy compared to other commodity resin grades [13]. Thus, it will be desirable if one can injection mold highly filled PC composites with reduced injection pressure in a consistent and reliable fashion.

Conventional injection molding (CIM) is one of the most versatile manufacturing methods for mass-producing plastic components with complex geometry, detailed surface replication, high production rates, and precise dimensions [14]. Successful processing of highly viscous polymers such as highly filled PC composites using CIM requires advanced molding skills and proper approaches. Using high processing temperatures and high injection rates can easily lead to material degradation, filling problems, and low-quality parts [15,16,17], adversely offsetting various key benefits of injection molded PC composites.

Microcellular injection molding (MIM) is an emerging process that uses physical foaming agents instead of chemical foaming agents for many foaming applications [18,19,20]. In MIM, atmospheric gases such as nitrogen or carbon dioxide in their supercritical fluid (SCF) phase are injected directly into the plasticizing injection barrel. During injection molding, the sudden pressure and temperature changes trigger the cell nucleation, and the dissolved gas emerges as numerous micro-scaled cells (bubbles) that help to fill the mold cavity. The continued cell growth from internal gas pressure can compensate for the volumetric shrinkage of the polymer, thereby eliminating the need for the pack/hold stage while improving the dimensional stability of the molded parts [21]. In addition, the plasticizing effect of the SCF and the uniform internal packing pressure from cell growth allow engineers to design parts with both thin and thick sections, leading to greater design freedom and part rigidity. Besides light-weighting and the part design freedom, many other advantages of MIM, such as reduced injection pressure, decreased thermal degradation, better mold filling, and reduced energy and overall cost, have been reported [22,23,24,25].

It is known that polymers with fine fillers tend to create quality foams since the fillers act as effective cell nucleating agents [24,25,26]. Previous studies show that the MIM processing of fiber-reinforced polymers creates cells preferentially in the matrix rather than on the fiber surface [27,28]. Therefore, it is expected that MIM will be a suitable and promising process for composites with both fibers and fillers. However, to the best of our knowledge, no study in the literature investigates MIM processing on highly filled PC composites loaded with glass fibers and fine carbon black powders.

The objective of this study is to investigate the processability of such a PC composite using both CIM and MIM. The work aims to show how different processing strategies of MIM will affect foam morphology and part quality. Herein, injection pressure requirements, part weight reduction, surface characteristics, SEM images of microstructure, and mechanical properties of the solid and foamed tensile test bars and box-shaped samples will be presented.

## 2. Materials and Experiments

A highly filled polycarbonate (PC) composite, Carbo-Rite series from Lubrizol Advanced Materials Company (Wickliffe, OH, USA) with 15% glass fibers and 20% carbon black by weight was used in this study [29]. As recommended by the resin manufacturer, the PC composite was dried in a vacuum oven at 125 °C for 5 h before injection molding. An Arburg Allrounder (Arburg, Lossburg, Germany) 320S injection molding machine with a clamping capacity of 500 kN and an SCF injection unit from Trexel (Wilmington, MA, USA) was used in this study.

### 2.1. Conventional and Microcellular Injection Molding of ASTM Tensile Test Bar Samples

ASTM D638-03 Type I tensile test bar samples from both of the conventional injection molding (CIM) and microcellular injection molding (MIM) processes were collected after the machine was run for at least 15 cycles and reached a stable molding condition. For each sample type, at least five samples were randomly selected for the weight measurements. Furthermore, tensile testing of the molded samples was performed. A minimum of five samples of each sample type were tested with a crosshead speed of 5 mm/min as recommended by the standard.

Randomly selected test samples for SEM imaging were cryogenically fractured by immersion in liquid nitrogen. Each sample was then coated with 5 nm of platinum by sputtering. The NeoScope JCM-5000 scanning electron microscope (SEM) from JEOL, Ltd. (Tokyo, Japan) was used to examine the microstructure of the fractured surfaces of the samples.

Table 1 lists the various molding trials and sample types together with the corresponding processing parameters used. For the solid molding trial, the processing parameters were selected in such a way that solid tensile test bar samples could be produced consistently and continuously. Three different MIM trials were conducted to produce samples with different part weights and microcellular structures. In particular, MIM-1 samples were molded with processing parameters similar to those of the solid samples except that the back pressure used was higher and there was no pack/hold stage in MIM. Subsequently, the processing parameters were adjusted to produce samples with better foam morphology in the MIM-2 trial and samples with a greater weight reduction in the MIM-3 trial, respectively. Supercritical nitrogen (scN_2_) was employed in all the MIM trials as it typically yields a finer-foamed structure when compared to supercritical carbon dioxide (scCO_2_) for injection molded parts.

### 2.2. Solid and Microcellular Injection Molding of Box-Shaped Samples

**Figure 1:**
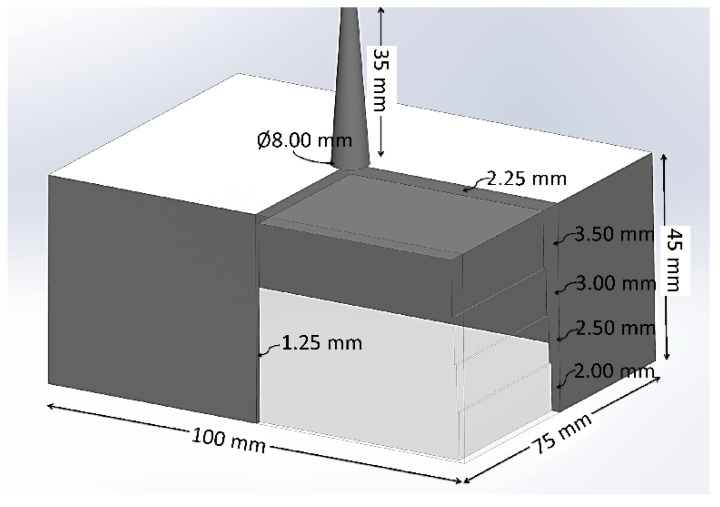
3D model of the box-shaped part. A quarter of the part is cut away to show the variable wall thickness.

Both CIM and MIM were employed to mold more complex, box-shaped parts to examine the process stability, part consistency, weight reduction, and injection pressure requirement. Figure 1 shows a 3D model of the box-shaped part with overall dimensions. Intended to produce molded parts with severe shrinkage and warpage for research purposes, the design of the box-shaped part was intentionally flawed, with sharp corners, lack of cooling channels in the core, and a step-wise decreasing thickness (from 3.5 to 2.0 mm in steps of 0.5 mm) on the shorter wall [21]. The wider wall had a smaller thickness of 1.25 mm. The thickness of the top wall was 2.25 mm. The variable wall thickness and abrupt changes in thickness would cause the material to race-track, thereby presenting a difficult-to-mold case for both CIM and MIM.

Table 2 tabulates the corresponding process and the processing parameters used for molding the box-shaped parts. As before, supercritical nitrogen (scN_2_) was employed in all MIM trials. More than 10 parts were collected after the process reached its steady-state condition. Parts were weighted to obtain the average part weight, and the injection pressure at 90% cavity fill was recorded to compare the required injection pressure between CIM and MIM. Gate dimensions should take into account the effect of the pressure requirement and material used. A sprue gate with a diameter of 8 mm was used for the box-shape mold, and a 2 by 4 mm gate and a 6 mm diameter runner were used for the tensile test bar mold.

## 3. Results

### 3.1. Weight Reduction and Injection Pressure of Tensile Test Bar Samples

**Figure 2:**
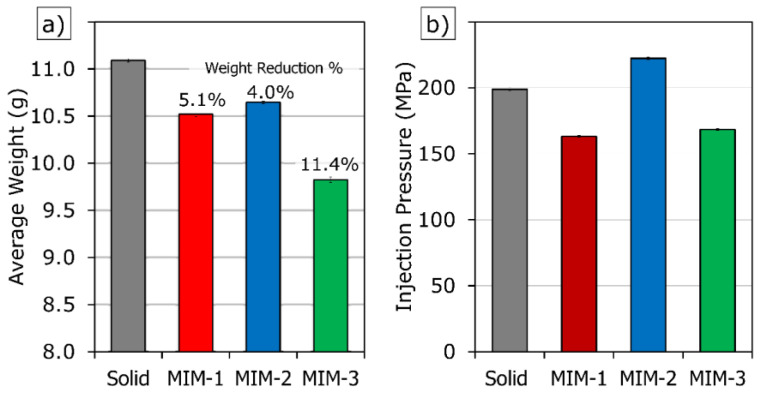
(**a**) Average part weight; (**b**) injection pressure values of tensile test bar samples.

Injection pressure is one of the most important process parameters in injection molding for purposes of process monitoring, control, and cost estimate. A high injection pressure causes a cost increase by reducing machine and mold life and directly increases the energy cost per part in a production where efficiency is critical. It should also be noted that the high pressure in injection molding is an obstacle to robust and reliable production. Therefore, it is important to consider the injection pressure for the highly filled PC composites. Figure 2 shows the average part weight and maximum injection pressure values of the four tensile test bar sample types, i.e., Solid, MIM-1, MIM-2, and MIM-3. Foamed samples (all MIMs) were consistently lighter than the solid parts due to the absence of the pack/hold stage even though the same shot volume was used for Solid, MIM-1, and MIM-2 during mold filling. The actual weight reductions were 5.1% for MIM-1, 4.0% for MIM-2, and 11.4% for MIM-3 samples, respectively. The small standard deviations of average weights suggested that the weight reduction of the samples can be adjusted easily and consistently for reliable foaming of this highly filled PC composite.

The average injection pressure of the various samples can be seen in Figure 2b. The injection pressures for all the samples were relatively high compared to most of the commodity resins. This was due to the very high filler content and the high melt viscosity of PC. Another reason for the high injection pressure might be due to the processing temperature (i.e., the melt temperature) setting being slightly lower than typical. It is useful to keep the temperature value low, even if it will increase the injection pressure to a certain extent, as increasing the melt temperature could lead to a coarse cell structure in the foamed parts.

In general, the injection pressure can be affected by melt and mold temperatures, injection speed, and shot volume. Another parameter that can influence the injection pressure is the dissolved gas content (i.e., gas dosage) in the polymer melt for MIM. Recall that the supercritical fluid (SCF) used in MIM could reduce the injection pressure due to the plasticizing effect [22]. The average injection pressure of the MIM-1 trial was 18% lower than that of the solid trial (cf. Figure 2b), even though these two trials employed the same injection and temperature settings. However, the average injection pressure of the MIM-2 trial was 12% higher than that of the solid trial. This increase was due to the increased injection speed and decreased temperature setting of the MIM-2 trial. Compared to the MIM-1 trial, the MIM-3 trial required a lower injection pressure since less polymer was injected into the cavity. Shot volume had a greater effect on the injection pressure of the MIM-3 trial than the slight increase in the injection speed (cf. Table 1).

### 3.2. SEM Images of Tensile Test Bar Samples

**Figure 3:**
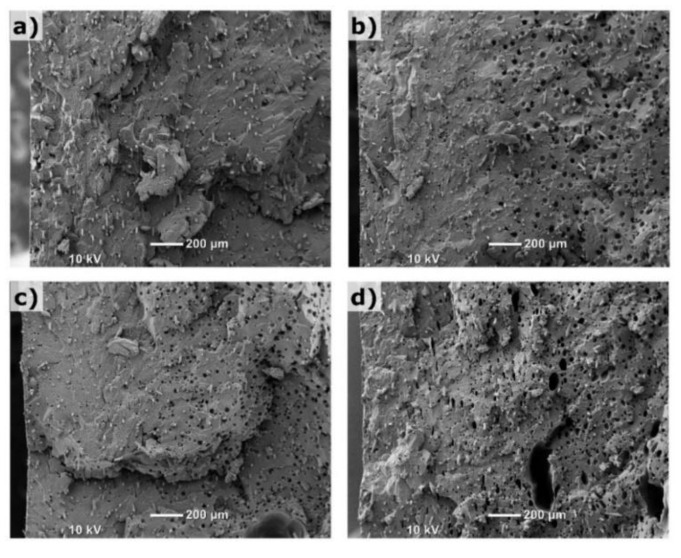
SEM images of cryogenically fractured tensile test bar samples at lower magnification: (**a**) solid; (**b**) MIM-1; (**c**) MIM-2; (**d**) MIM-3.

Figure 3 shows the SEM images of cryogenically fractured surfaces of the tensile test bar samples. On the left side of the SEM images, the part surface could be clearly seen. The centers of the fractured samples were approximately located on the right edge of the images. The flow direction was into the page. The glass fibers and the typical holes from pulled-out fibers could be seen on the fractured surface of the solid sample in Figure 3a. These fiber holes could be distinguished from foaming cells by their smaller size and deep tubular shape in the foamed samples.

**Figure 4:**
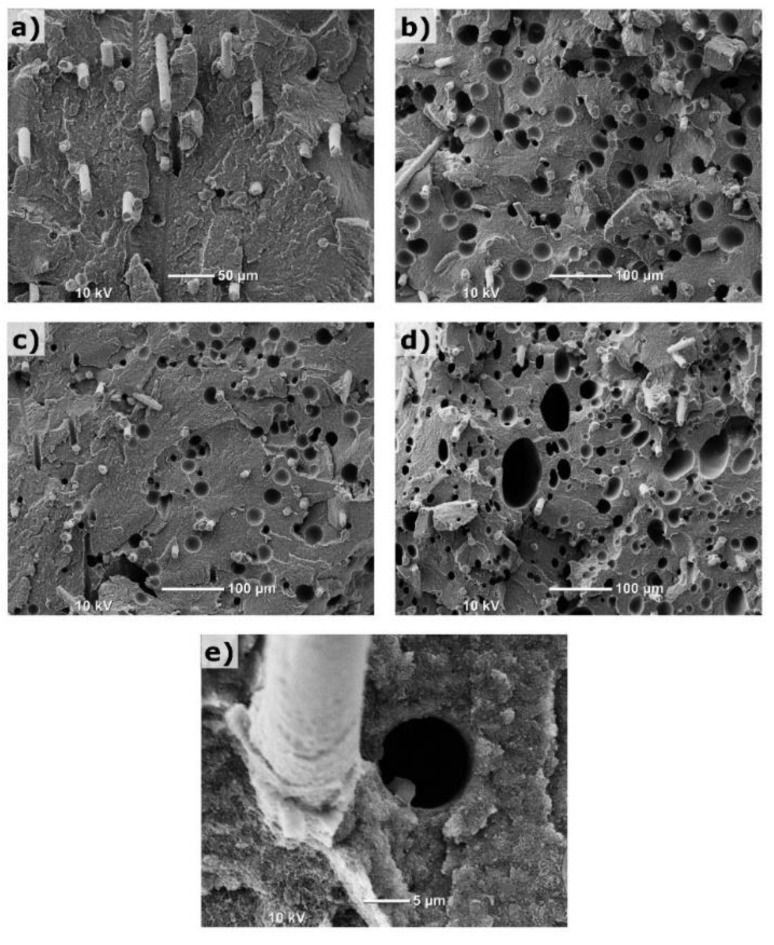
SEM images of molded samples at higher magnification: (**a**) solid; (**b**) MIM-1; (**c**) MIM-2; (**d**) MIM-3; (**e**) a fiber and a fiber hole left by a pulled-out fiber.

Overall, the foamed samples have a relatively uniform cell structure. The optimized processing parameters used in the MIM-2 trial improved the cell morphology, leading to a smaller cell size and denser cell structure. In general, increasing the weight reduction could adversely affect the cell structure in the foamed parts. For example, the MIM-3 sample had some large cells despite a relatively fine foamed structure for the rest of the cross section. It was found that the processing parameters associated with the MIM-3 trial did not increase the average cell size. Instead, they generated some large cells, which could be associated with a higher dosage of gas. It should be noted that the SCF could only be dissolved in the polymer matrix and not in the solid fillers. Therefore, the weight percent of dissolved gas in the matrix polymer was actually higher than that listed in Table 1, which was based on the total part weight of the solid part. The SEM images with a higher magnification were taken to examine the fine details of the foam morphology, as shown in Figure 4.

Carbon black particles are not visible in any of the samples due to their fine sizes. The SEM images showed that most of the nucleation points were within the polymer matrix instead of being on the fiber surfaces. This confirms that MIM is suitable for molding highly filled PC composites with glass fibers and carbon black as the foaming will not reduce the polymer/fiber interface areas or hinder the fiber reinforcement effect. Figure 4e shows a fiber and a fiber hole left by a pulled-out fiber at a higher magnification. As mentioned above, it could be distinguished from typically larger foaming cells.

### 3.3. Tensile Test Bar Sample Images

**Figure 5:**
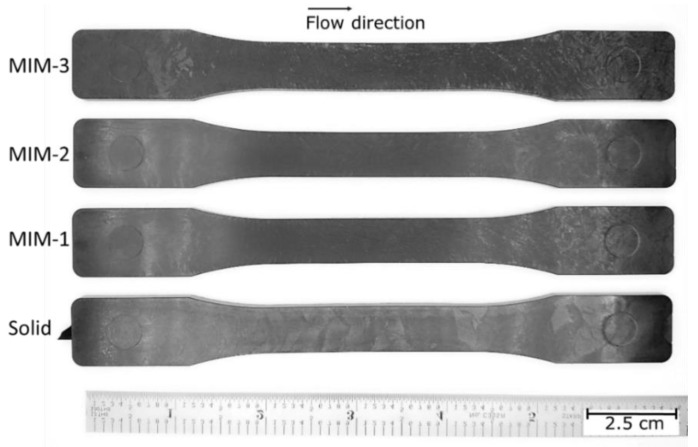
Surface characteristics of various molded samples.

Figure 5 shows a part photo for each tensile test bar sample type and their surface characteristics. The foaming that occurs on the melt front during the filling is known to cause some swirl marks on the part surface [30]. It can be observed that MIM samples are darker in color due to the fact that swirl marks increased the roughness of the surfaces. It was noted that the MIM-3 sample exhibited the most noticeable swirl marks, especially near the end of the fill (away from the gate), as compared to the MIM-1 and MIM-2 samples. This was because the MIM-3 sample had a higher gas dosage and a smaller shot volume, which led to greater weight reduction. One can see that the MIM-2 sample had fewer visible swirl marks compared to the MIM-1 sample. This suggested that processing parameters such as a larger shot volume, higher melt and mold wall temperatures, and lower gas dosage helped to reduce the swirl marks.

**Figure 6:**
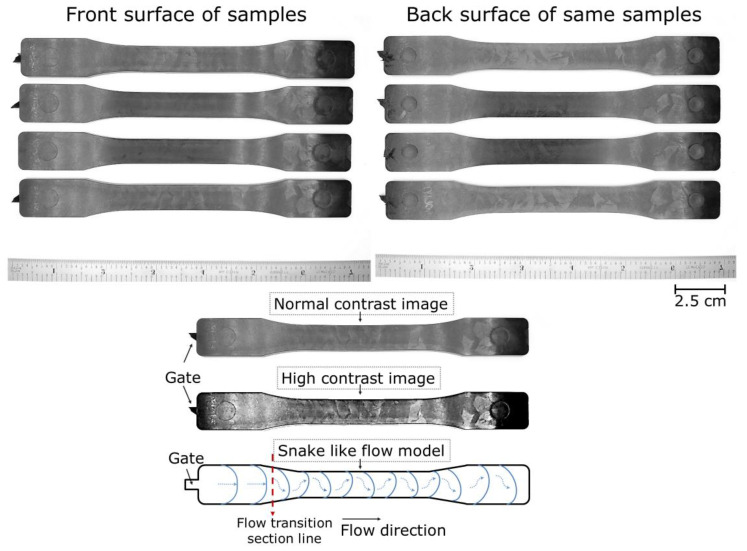
(**Top**) Front and back surfaces of four randomly selected solid samples. (**Bottom**) The speculated melt flow and transition model responsible for the irregular tiger-stripes pattern.

Figure 6 shows the front and back surfaces of four randomly selected solid samples. The surfaces of all the solid samples had the same unique patterns, consisting of both smooth and dull regions, which is not as visible on the foamed MIM samples. This type of pattern could be attributed to the oscillating or snake-like melt front advancements that led to the so-called tiger stripes [31]. This surface feature that consists of alternate smooth and dull regions is known to be more common for polymer composites with a solid or rubber filler [32].

A high contrast image (cf. Figure 6 bottom) was created to show the glossy and cloudy regions on the part surface. The gate side of the solid samples exhibited more even and parallel tiger stripes compared to the rest of the sample surfaces. This suggested that the melt front started to oscillate after it reached the contracting region of the dog-bone shape cavity [32]. A speculated melt flow and transition model as shown in Figure 6 was proposed to illustrate the forming mechanism of such an irregular tiger-stripes pattern.

### 3.4. Mechanical Properties of Tensile Test Bar Samples

**Figure 7:**
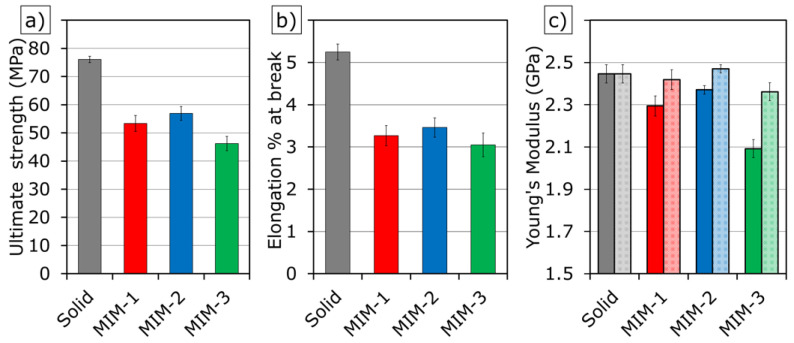
Mechanical properties of tensile test bar samples: (**a**) ultimate strength; (**b**) elongation at break of samples; (**c**) Young’s Modulus and specific Young’s modulus of samples. Bars with a textured pattern show the specific Young’s modulus.

The foaming reduced the ultimate strength of the MIM samples, as seen in Figure 7a. This reduction could be associated with the weight reduction, reduced effective cross-sectional area due to the presence of cells, and stress concentrations at the cell walls. The results showed that increasing the weight reduction tended to worsen the ultimate strength, while optimization of cell structure could improve it. Although MIM negatively affected the mechanical property of this PC composite, it still retained enough strength while reducing the part weight and material consumption. Considering the effects of light-weighting, the specific Young’s modulus (the measured Young’s modulus divided by the weight ratio of foamed to solid samples) of four sample types were compared, as shown in Figure 7c. In this study, it was found that the foamed parts had only a slight decrease in the specific Young’s modulus when compared to the solid samples. This showed that most of the decrease in mechanical properties seen in the foamed parts was due to the reduction in part weight.

**Figure 8:**
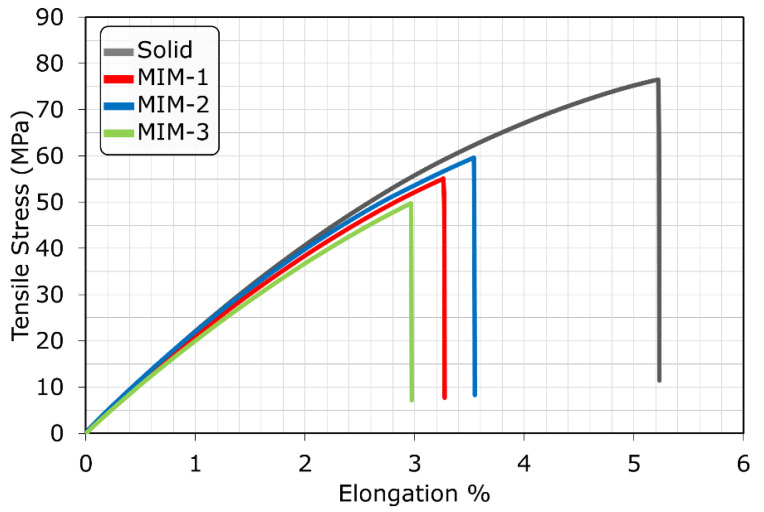
Tensile stress vs. strain (elongation %) curves for the four tensile test bar samples.

Interestingly, within the weight reduction range of this study, the elongation at break among the MIM tensile test bar samples seemed to be independent of part weight reduction, as shown in Figure 7b. This indicated that there was a critical strain on the cell surface that initiated the fracture, which was independent of weight reduction. Figure 8 shows the representative tensile stress vs. strain (elongation %) curves of each sample type. All sample types exhibited brittle characteristics and a lack of plastic deformation due to the heavy loading (35 wt%) of fillers in the PC composite.

### 3.5. Comparison of Required Injection Pressure for the Box-Shaped Part

**Figure 9:**
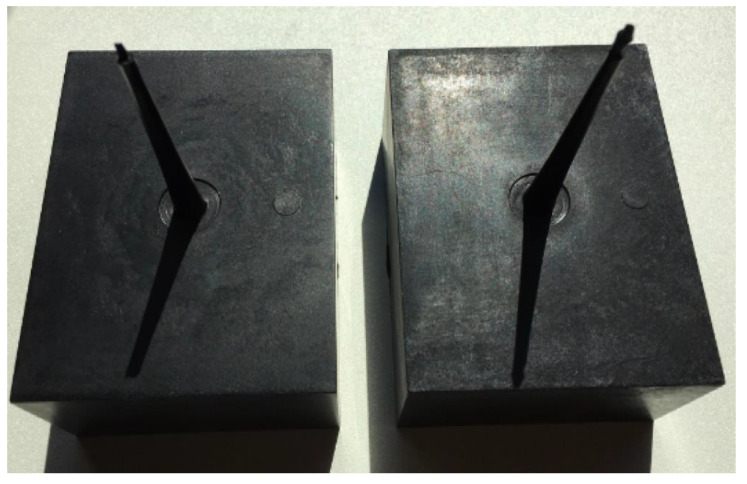
(**Left**) solid and (**right**) MIM box-shaped parts.

Figure 9 shows the box-shaped parts molded by both of the CIM and MIM processes. All the foamed box-shaped parts seemed identical and were free of any large cells or blisters on part surfaces. As shown in Table 3, a nearly 26% injection pressure reduction could be achieved with an average weight reduction of 5.23%. The pressure reduction was consistent for the entire MIM molding trial when compared with the CIM experiment.

It was interesting to observe that the injection pressure required to produce the box-shaped parts (102.6 MPa) was about half of that for the tensile bar samples (198.8 MPa). This may seem contradictory at first, as the box-shaped part has a much larger shot volume and thinner sections. However, it is necessary to look at the flow length-to-thickness ratio of the mold samples as a proper measure for comparing the injection pressure requirements. Flow length-to-thickness ratio can be simply calculated by dividing the longest path traveled by the melt to the average section thickness. Since the wall thickness of the box-shaped part is not constant, the flow length-to-thickness ratio was calculated using an average wall thickness. Based on the mold runner and cavity dimensions, the flow length-to-thickness ratio was 121 for the tensile bar mold and 66 for the box-shaped mold. In light of the similar ratio of injection pressure (198.8/102.6 = 1.94) and the flow length-to-thickness ratio (121/66 = 1.83), it could be concluded that the moldability of this highly-filed PC composite could be estimated by the flow length-to-thickness ratio for the different mold designs. In addition, it can be seen that the gate area for the box-shaped part (50 mm^2^) is larger than the gate area of the tensile bar part (8 mm^2^). This could be the other reason for the injection pressure difference.

## 4. Conclusions

A highly filled PC-based composite with glass fibers and carbon black was processed by both CIM and MIM processes. The results showed that the SCF used for the MIM process successfully reduced the required injection pressure for both the tensile test bars and the complex box-shaped parts. This was a significant achievement for this difficult-to-process material and difficult-to-mold box-shaped part. As could be seen from the SEM images, the foamed morphology of the tensile test bar samples had relatively fine and regular cells. Although the MIM process caused a slight decrease in tensile properties, the specific Young’s modulus was comparable to that of the solid samples. Nonetheless, the slight material property reduction could be easily compensated by creative and flexible part geometry designs, a major benefit offered by MIM. In addition, the unique, irregular tiger-stripes surface pattern with glossy and cloudy regions on the solid samples was observed and their causes were illustrated by a speculated melt flow and transition model. Further, the MIM process is shown to reduce this surface defect. Finally, MIM could be used to produce a complex, box-shaped part consistently with 5.23% reduction in part weight and 25.8% reduction in injection pressure when compared to CIM.

## Figures and Tables

**Table 1:** Four molding trials and sample types as well as the corresponding processing parameters. Up and down arrows indicate the adjustment compared to the solid injection molding setting or MIM-1.

Parameters	Unit	Solid	MIM-1	MIM-2	MIM-3
Injection speed	cm^3^/s	30	30	↑50	↑40
Shot volume	cm^3^	21	21	21	↓18
Packing pressure	MPa	80	N/A	N/A	N/A
Packing time	s	8	N/A	N/A	N/A
Back pressure	MPa	0.2	5.0	5.0	5.0
Melt temperature	°C	300	300	↓285	↑310
Mold temperature	°C	80	80	↓60	↑90
Cooling time	s	20	20	20	↑25
SCF (N_2_) dosage ^1^	wt%	N/A	0.50	↑0.55	↑0.61
Actual weight reduction	wt%	N/A	5.1	↓4.0	↑11.4

^1^ N_2_ % dosage was based on the total part weight of the solid part.

**Table 2:** Processing parameters for solid CIM and MIM box-shaped parts.

Sample Name		Solid	MIM
Parameters	Unit	Value	Value
Injection speed	cm^3^/s	30	30
Injection volume	cm^3^	52.0	51.8
Packing pressure	MPa	80	N/A
Packing time	s	8	N/A
Back pressure	MPa	0.2	5.0
Melt temperature	°C	320	320
Mold temperature	°C	80	80
Cooling time	s	30	30
SCF (N_2_) dosage ^1^	wt%	N/A	0.50
Nominal weight Reduction	wt%	N/A	5

^1^ N_2_ % dosage was based on the total part weight of the solid part.

**Table 3:** Injection pressure and weight comparison.

Sample Name		Solid	MIM	Solid and MIM Comparison
Parameters	Unit	Value	Value	% Change
Average weight	g	59.41 ± 0.10	56.30 ± 0.24	5.23
Injection pressure at 90% of filling	MPa	102.6 ± 2.2	76.2 ± 2.4	25.8

## Data Availability

Not applicable.
